# Doctor, Will My Surgical Hardware Set Off Metal Detector in the Airport?

**DOI:** 10.1055/s-0043-1771493

**Published:** 2023-10-24

**Authors:** Igor Guedes Nogueira Reis, Beatriz Marinho Guimarães, Samuel Henrique Ferreira de Souza, Marco Antônio Percope de Andrade, Robinson Esteves Pires

**Affiliations:** 1 Residência em Cirurgia Ortopédica, Departamento do Aparelho Locomotor, Hospital das Clínicas, Universidade Federal de Minas Gerais, Belo Horizonte, MG, Brasil; 2 Departamento de Aparelho Locomotor, Hospital das Clínicas, Universidade Federal de Minas Gerais, Belo Horizonte, MG, Brasil

**Keywords:** airports, internal fracture fixation, metals, patient education as topic, prosthesis and implants

## Abstract

**Objective** Verify if routinely used metallic implants (stainless steel, aluminum alloy, cobalt-chromium-molybdenum, and titanium made) will be detected in an international airport of Brazil and generate helpful information to prevent patient inconvenience and to support the security regulatory agencies.

**Methods** An experimental, non-randomized, controlled, cross-over study was performed by recruiting two individuals, one male and one female, to pass through a standard airport metal detector with orthopedic implants attached to the body. Implants with different compositions, weight, and in various parts of the body were tested.

**Results** From all implants tested, there was no detection of implants for internal fixation, whether steel or titanium. The external fixator was detected and the only difference in composition is that the external fixator tested have aluminum alloy. All hip replacement implants tested were detected. Two knee replacement implants were tested, and both were made of cobalt-chromium-molybdenum, but with different specifications and only one of them was detected.

**Conclusions** In this study with ex-vivo orthopedic implants, we have found that osteosynthesis implants composed by Stainless Steel ISO 5832-1 did not trigger the airport walk-through metal detector. However, external fixator and total joint prostheses were more frequently detected.

## Introduction

An agreement on what are the recommendations for patients with implants in regard to air travel still lacks in current literature. However, due to the catastrophic incident of September 11 ^th^ , 2001, data on this topic has arisen progressively. 1 2 3 4 This historical tragedy stimulated travel restrictions and sensitivity of airport metal detectors were increased, leading to inconvenience, such as thorough and prolonged body search reported by patients during national and international air travels. 5 6 7 8 9

It is still a matter of controversy the factors that influence detection of some implants, but some hypothesis were generated by observational studies, surveys and experimental studies. 4 Metal composition, metal weight, amount of body mass, distance from metal to detector, and speed when crossing the detector are potential elements that can influence the metal detection. It is noteworthy that we did not find any study on this topic in the Brazilian literature, which confirms that the topic is worthy of investigation.

Therefore, we have performed this experimental study to foment the discussion in the Brazilian scientific community, investigate possible factors associated to orthopedic implant detection in airports and generate useful data for a better guidance to patients and to the air-security agencies.

## Materials and Methods

Prior approval was obtained from the responsible research ethics committee in our institution (CAAE: 57064422.0.0000.5149) and from the airport security. Informed consent was obtained from participants. An experimental, non-randomized, controlled, cross-over study was carried out on August 12, 2022 at the Tancredo Neves International Airport in Belo Horizonte (MG), Brazil.

**Fig. 1:**
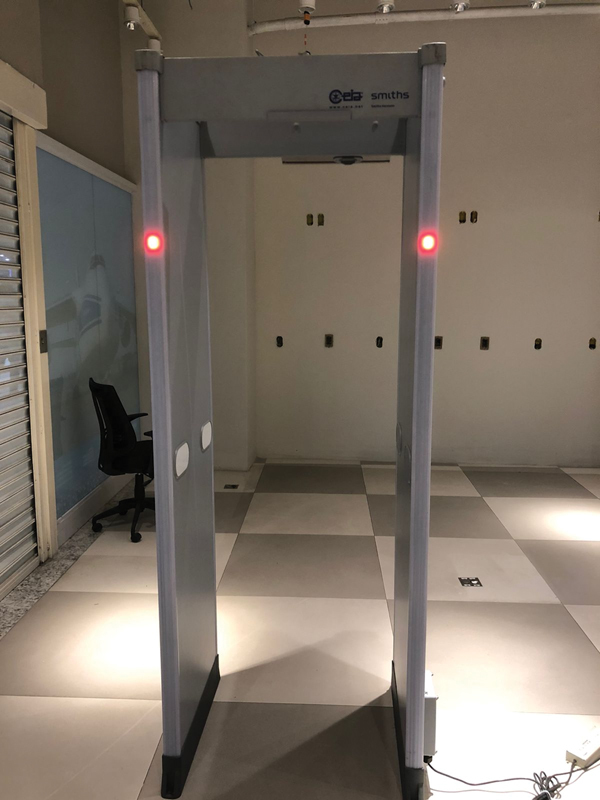
Airport walk-through metal detector (CEIA SMD600 PLUS, 2021 model).

**Fig. 2:**
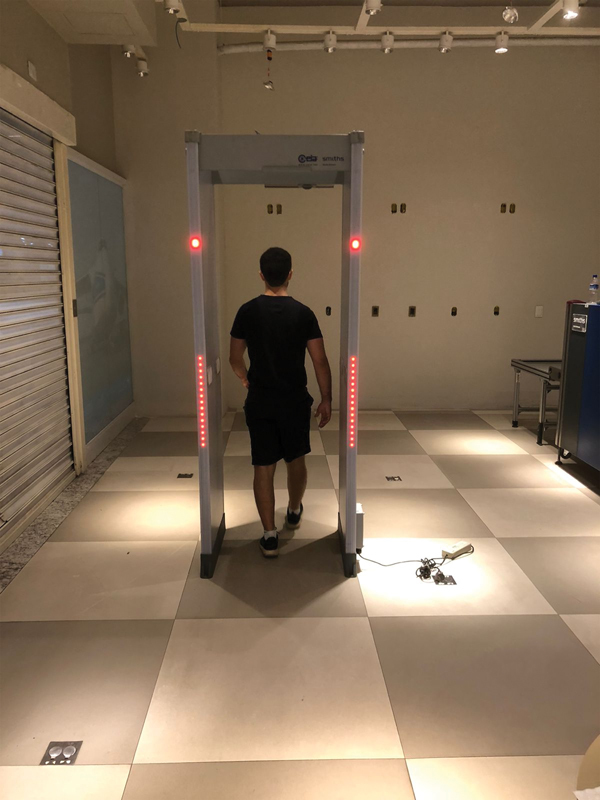
Control test to certify appropriate functioning of the arch detector using cellphone in the pocket.

The airport walk-through metal detector ( Fig. 1 ) used was CEIA SMD600 PLUS (2021 model), device universally used in international airports. The experiment was performed in a device set for regular airport daily routine. Before starting, we made sure the device was working appropriately ( Fig. 2 ) by testing with metallic objects (belts and cellphones) and, at all times, the tests were performed under supervision of the responsible technician to ensure perfect functioning.

**Fig. 3:**
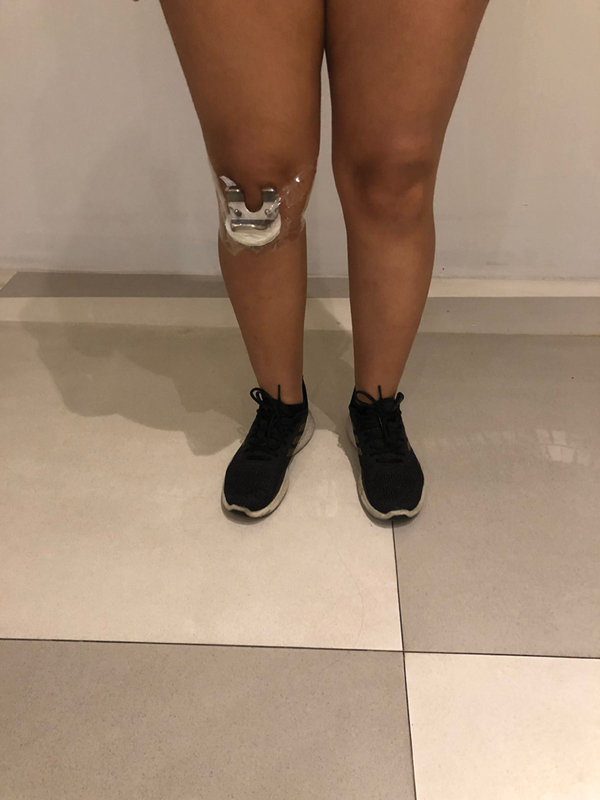
Total knee prosthesis strapped to the anterior surface of the right knee before testing.

**Fig. 4:**
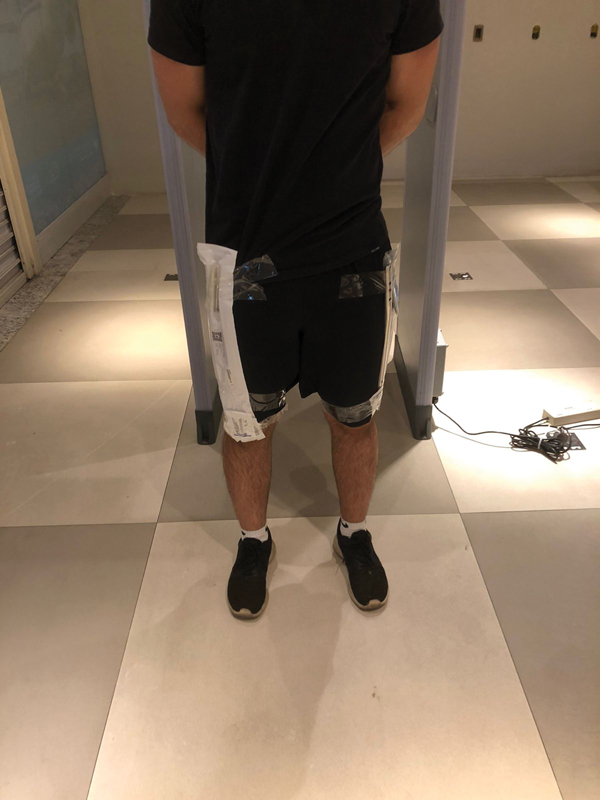
Bilateral retrograde femoral nail strapped to the lateral surface of the thighs before testing.

Two healthy volunteers, one male (172 cm in height) and one female (156 cm in height), who had no metal device in their body, were recruited. The volunteers walked in two different speeds each, one test at 2 km/hr and one at 6 km/hr. Initially, as a control, the volunteers walked across the metal detector without implants. Afterwards, we strapped orthopedic implants to the volunteers ( Figs. 3 and 4 ), using an adhesive tape, respecting as much as possible the true location of the implant in the body. All the tests were performed twice to ratify the results of the first pass.

Implants ( Table 1 ) used for fracture fixation and for joint replacement were assessed in different combinations (unilateral/bilateral, left/right limbs or associated fractures) and always respecting the anatomic location (shoulder, arm, elbow, forearm, wrist, hip, thigh, knee, leg, and ankle). The implants for internal fixation were manufactured by Hexagon ^®^ (Itapira, SP, Brazil), Smith & Nephew (Memphis, TN, USA) and Tóride (Mogi Mirim, SP, Brazil), the external fixator by Baumer (Mogi Mirim, SP, Brazil), the hip prostheses by Baumer (Mogi Mirim, SP, Brazil) and Víncula (Rio Claro, SP, Brazil), and the knee prostheses by Aesculap AG (Tuttlingen, Alemanha) and Baumer (Mogi Mirim, SP, Brazil).

**Table 1:** Orthopedic implants sets and combinations tested

Implant	Male volunteer				Female volunteer				Alloy
	2 km/h		6 km/h		2 km/h		6 km/h		
	Test 1	Test 2	Test 1	Test 2	Test 1	Test 2	Test 1	Test 2	
Cellphone (control)	P	P	P	P	P	P	P	P	–
None (control)	N	N	N	N	N	N	N	N	–
Small fragment one-third tubular plate + 6 screws (unilateral)	N	N	N	N	N	N	N	N	Stainless steel NBR ISO 5832-1
Medial distal tibial locking compression plate + 6 screws (unilateral)	N	N	N	N	N	N	N	N	Stainless steel NBR ISO 5832-1
Medial and anterolateral distal tibial locking compression plate + 12 screws (unilateral)	N	N	N	N	N	N	N	N	Stainless steel NBR ISO 5832-1
Small fragment calcaneus plate + 6 screws (unilateral)	N	N	N	N	N	N	N	N	Stainless steel NBR ISO 5832-1
Intramedullary tibial nail + 4 locking screws (unilateral)	N	N	N	N	N	N	N	N	Stainless steel NBR ISO 5832-1
Intramedullary retrograde femoral nail + 3 locking screws (unilateral)	N	N	N	N	N	N	N	N	Stainless steel NBR ISO 5832-1
Intramedullary retrograde femoral nail + 3 locking screws (bilateral)	N	N	N	N	N	N	N	N	Stainless steel NBR ISO 5832-1
Bilateral intramedullary retrograde femoral nail + 6 locking screws + unilateral intramedullary tibial nail + 3 screws	N	N	N	N	N	N	N	N	Stainless steel NBR ISO 5832-1
Cephalomedullary femoral nail + 1 sliding hip screw + 2 locking screws (unilateral)	N	N	N	N	N	N	N	N	Titanium Ti-6Al-4V (ASTM F1472)
Cephalomedullary femoral nail + 1 sliding hip screw + 2 locking screws (bilateral)	N	N	N	N	N	N	N	N	Titanium Ti-6Al-4V (ASTM F1472)
DCS with screws(unilateral)	N	N	N	N	N	N	N	N	Stainless steel NBR ISO 5832-1
DHS with screws (unilateral)	N	N	N	N	N	N	N	N	Stainless steel NBR ISO 5832-1
Proximal humeral locking compression plate + 8 screws (unilateral)	N	N	N	N	N	N	N	N	Stainless steel NBR ISO 5832-1
Two 3.5 mm anchor screws on the shoulder (unilateral)	N	N	N	N	N	N	N	N	Stainless steel NBR ISO 5832-1
Intramedullary humeral nail + 3 locking screws (unilateral)	N	N	N	N	N	N	N	N	Stainless steel NBR ISO 5832-1
Clavicle locking compression plate + 6 screws (unilateral)	N	N	N	N	N	N	N	N	Stainless steel NBR ISO 5832-1
Clavicle locking compression plate + 6 screws (bilateral)	N	N	N	N	N	N	N	N	Stainless steel NBR ISO 5832-1
12 holes DCP + 4 screws (unilateral humerus)	N	N	N	N	N	N	N	N	Stainless steel NBR ISO 5832-1
Two 3.5 mm cannulated screws + cerclage wire (1.0 mm) (unilateral knee)	N	N	N	N	N	N	N	N	Stainless steel NBR ISO 5832-1
Two 3.5 mm cannulated screws + cerclage wire (1.0 mm) (bilateral knee)	N	N	N	N	N	N	N	N	Stainless steel NBR ISO 5832-1
Two 7.0 mm cannulated screws + 1 cerclage wire (1.0 mm) (unilateral knee)	N	N	N	N	N	N	N	N	Stainless steel NBR ISO 5832-1
Two 7.0 mm cannulated screws + 1 cerclage wire (1.0 mm) (bilateral knee)	N	N	N	N	N	N	N	N	Stainless steel NBR ISO 5832-1
Distal radius locking compression plate + 11 screws (unilateral)	N	N	N	N	N	N	N	N	Stainless steel NBR ISO 5832-1
Distal radius locking compression plate + 11 screws (bilateral)	N	N	N	N	N	N	N	N	Stainless steel NBR ISO 5832-1
Distal radius locking compression plate + 11 screws (unilateral)	N	N	N	N	N	N	N	N	Titanium (ASTM F-67)
Distal radius locking compression plate + 11 screws (bilateral)	N	N	N	N	N	N	N	N	Titanium (ASTM F-67)
Cemented primary total knee prosthesis (unilateral)	P	P	P	P	P	P	P	P	Cobalt-chromium-molybdenum (ISO 5832-4)
Cemented primary total knee prosthesis (unilateral)	N	N	N	N	N	N	N	N	Cobalt-chromium-molybdenum (ISO 5832-4)
Thompson hip prosthesis (unilateral)	P	P	P	P	P	P	P	P	Stainless steel NBR ISO 5832-1
Uncemented acetabulum + uncemented femoral stem (unilateral)	P	P	P	P	P	P	P	P	Titanium (ASTM F-67) (acetabulum) + Titanium Ti-6A-4V (ASTM F-136) and titanium porous coating (ASTM F-1580) (femoral component)
Uncemented acetabulum + cemented primary femoral stem (unilateral)	P	P	P	P	P	P	P	P	Titanium (ASTM F-67) (acetabulum) + Stainless steel (NBR ISO 5832-9/ASTM F-1586)
External fixator (4 Schanz screws + 2 bars + 8 self-holding clamps)	P	P	P	P	P	P	P	P	Stainless steel (ISO 5832-1, ASTM F-138) and aluminum alloy

Abbreviations: ASTM, American Society for Testing and Materials; N, negative; NBR ISO, Norma Brasileira Regulamentadora - International Organization for Standardization; P, positive.

## Results

The results are presented in Table 1 . From all osteosynthesis devices tested, the external fixator was the only one detected and its difference from the other devices was in its composition – it was made of aluminum alloy. All hip replacement implants tested were detected. Two knee replacement implants were tested, and both were made of cobalt-chromium-molybdenum, but with different specifications ( Table 1 ) and, only one of them was detected.

## Discussion

Patients frequently ask about practical aspects of daily living and how certain types of surgery will affect their routine and for how long. Many of these questions still do not have a definite answer based on scientific studies. An extremely common question is if retained orthopedic implants will trigger airport walk-through metal detectors. This has been a hot topic since September 11, 2001, when the World was shocked by airplanes terrorist attacks, after which airport security has increased to prevent such acts. The main concerns usually are the inconvenient of being body searched after triggering the metal detector, anxiety about being detained at the airport, and the possibility of delaying a travel or losing a flight. After 21 years of the terrorist attacks, to the best of our knowledge, there is still no official and universally accepted document that the patient can carry to prove the existence of an orthopedic metallic implant. Therefore, the standard procedure is further screening if an individual triggers a walk-through metal detector despite carrying a medical report issued by the orthopedic surgeon. 10 In Brazil, this is the first study to investigate orthopedic implant detection by airport walk-through metal detector. Additionally, all of the existing published researches were performed in arch detectors with more than 5 years of manufacture, while in this study, a 2021 device, universally adopted in international airports, was used. It is noteworthy that this metal detector fully complies the current security level of the Tancredo Neves International Airport (Confins), under regulation of the ANAC (National Agency of Civil Aviation), and consequently of the International Civil Aviation Organization.

Our findings were completely unexpected, as none of the osteosynthesis sets were detected, except the external fixator device. No differences were observed between the two distinct transit speeds assessed. All tests were performed with implants on both right and left sides, to minimize the potential bias of the distance from the metal to the detector, and no differences were observed. All osteosynthesis sets tested were made of Stainless Steel NBR ISO 5832-1, titanium F-67 (distal radius plate) and titanium Ti-6Al-4V (Cephalomedullary femoral nail), except the external fixator which had aluminum alloy in its composition. The only Stainless Steel NBR ISO 5832-1 implant detected was the Thompson hip prosthesis, which suggests that implant mass concentration might increase detection. All other types of hip prosthesis tested also triggered the alarm. An interesting finding was the difference in detection between the two types of total knee prosthesis tested. Although both knee prostheses were made of cobalt-chromium-molybdenum, the detected one was manufactured in Brazil, while the other one in Germany. This difference among the knee prostheses tested raise a suspicion about the composition of the alloy, which could possibly have interfered on the arch detector triggering.

Considering all combinations of implants tested and presented in Table 1 , our study corroborates with the findings of Chan et al., 11 in which all patients with foot and ankle implants alone passed undetected. This study is consonant with several others of the literature, which state that total joint prostheses will be frequently detected, such as the hip prostheses in our study. 2 3 12 Kimura et al. 5 found that implant detection rate was higher during international flights, which might explain why all hip prostheses were detected in our study, since the device used was set for international standards, probably a more sensitive configuration.

Previous researches suggested some factors that might influence the probability of detection, such as implant mass, combinations, composition, location within the body, laterality, transit speed, detector model, sensitivity settings according to the security level of the airport, and tissue masking. 4 8 11 In our study, we were only capable to observe the probable interference of implant mass, density and material.

An interesting information about airport walk-through metal detectors is the fact that they record every individual that passes throw the arch even if there is no metal device in situ. This data is used in a randomizing internal software, which triggers a sound alarm to select a random passenger to be thoroughly body searched by the airport security. This sound alarm is different from the standard sound triggered when a metallic device is identified. This mechanism of selecting random passengers to be searched might be the reason why some patients with orthopedic implants report being body searched, which is a potential bias of previous research using retrospective information. 8

The limitations of our study include the fact that none of the orthopedic implants were inside the volunteers tested and we still do not know if a bone-implant or soft tissues-implant interaction would affect detection. Previous researches tried to assess how the soft tissue envelope affect metal detection, however results were conflicting. 4 11 Another limitation is that we were not able to test all the available orthopedic implants. However, we judge that the most frequent situations were assessed. Our study also did not assess detection by portable wand metal detector, which is supposed to be more sensitive. Our findings were based on the current security level of the Confins International Airport. If, for some reason, the airport security level increases, the device's settings also change, and the detection sensitivity will improve. Although the tests were performed in a real environment of an international airport, following all the security requirements and under ANAC and ICAO rules, we cannot affirm that our findings will be faithfully reproducible if carried out at other airports, using a different detector device or a different security level.

However, some strengths deserve to be highlighted. This is the first study performed in Brazil aiming to clarify information regarding detection of orthopedic implants in an airport. We provided helpful information to patients, since the implants for internal fixation probably will not be detected in normal conditions. On the other hand, external fixators and prostheses will probably be detected. We also tried to minimize potential biases observed in similar studies. We tested different metal compositions in two healthy volunteers, under two different speeds to pass the detector arch, with implants attached in both sides (right and left) of the body, to minimize the bias of the distance from the detector to the implant.

## Conclusions

In this study with ex-vivo orthopedic implants, we verified that osteosynthesis implants for internal fixation, composed by Stainless Steel ISO 5832-1 and titanium, were not detected by the airport walk-through metal detector and should not cause inconvenient to patients while travelling. However, external fixators and total joint prostheses will more frequently be detected.

It is important to highlight the limitations of our study and the need to further investigate this matter including in-vivo orthopedic implants and testing prostheses with different sizes, weights and materials.
