# Supplementary material for: Doctor, Will My Surgical Hardware Set Off Metal Detector in the Airport?
Source: Rev Bras Ortop (Sao Paulo). 2023 Oct 24;59(5):e672–81. doi: 10.1055/s-0043-1771493 (PMC11624945; doi:10.1055/s-0043-1771493)
Supplement: Related texts

# Research article: Doctor, Will My Surgical Hardware Set Off Metal Detector in the Airport?

**Keywords:** aeroportos, educação de pacientes como assunto, fixação interna de fraturas, metais, próteses e implantes.

## Abstract

**Objetivo** Verificar se implantes metálicos de uso rotineiro (aço inoxidável, liga de alumínio, cobalto-cromo-molibdênio e feitos de titânio) serão detectados em um aeroporto internacional do Brasil e gerar informações úteis para evitar transtornos ao paciente e apoiar os órgãos reguladores de segurança.

**Métodos** Estudo experimental, não randomizado, controlado e cruzado foi realizado recrutando dois indivíduos, um homem e uma mulher, para passar por um detector de metais padrão do aeroporto com implantes ortopédicos presos ao corpo. Foram testados implantes com diferentes composições, pesos e em várias partes do corpo.

**Resultados** De todos os implantes testados, não houve detecção de implantes para fixação interna, sejam de aço ou de titânio. O fixador externo foi detectado e a única diferença na composição é que o fixador externo testado é de liga de alumínio. Todos os implantes de artroplastia do quadril testados foram detectados. Dois implantes de artroplastia do joelho foram testados, e ambos eram feitos de cobalto-cromo-molibdênio, mas com especificações diferentes, e apenas um deles foi detectado.

**Conclusões** Neste estudo com implantes ortopédicos ex-vivo, verificamos que os implantes de osteossíntese compostos por aço inoxidável ISO 5832-1 não acionaram o arco detector de metais do aeroporto. No entanto, o fixador externo e as próteses articulares totais foram mais frequentemente detectadas.

## Introdução

Ainda falta na literatura atual um consenso sobre quais são as recomendações para pacientes com implantes em relação às viagens aéreas. No entanto, devido ao catastrófico incidente de 11 de setembro de 2001, os dados sobre esse tópico surgiram progressivamente. 1 2 3 4 Essa tragédia histórica estimulou as restrições de viagens e aumentou a sensibilidade dos detectores de metais dos aeroportos, levando a transtornos, como a revista corporal minuciosa e prolongada relatada por pacientes durante viagens aéreas nacionais e internacionais. 5 6 7 8 9

Ainda não há consenso sobre os fatores que influenciam a detecção de alguns implantes, mas algumas hipóteses foram geradas por estudos observacionais, levantamentos e estudos experimentais. 4 A composição do metal, o peso do metal, a quantidade de massa corporal, a distância do metal ao detector e a velocidade ao cruzar o detector são elementos potenciais que podem influenciar a detecção de metais. Vale ressaltar que não encontramos nenhum estudo sobre esse tema na literatura brasileira, o que confirma que o tema merece ser investigado.

Portanto, realizamos este estudo experimental para fomentar a discussão na comunidade científica brasileira, investigar possíveis fatores associados à detecção de implantes ortopédicos em aeroportos e gerar dados úteis para uma melhor orientação aos pacientes e aos órgãos de segurança aérea.

## Materiais e métodos

Obteve-se a aprovação prévia do comitê de ética em pesquisa responsável de nossa instituição (CAAE: 57064422.0.0000.5149) e da segurança do aeroporto. O consentimento informado foi obtido dos participantes. Um estudo experimental, não randomizado, controlado e cruzado foi realizado em 12 de agosto de 2022 no Aeroporto Internacional Tancredo Neves em Belo Horizonte (MG), Brasil.

O detector de metais de passagem utilizado no aeroporto ( Fig. 1 ) foi o CEIA SMD600 PLUS (modelo 2021), dispositivo universalmente utilizado em aeroportos internacionais. O experimento foi realizado em um dispositivo configurado para a rotina diária regular do aeroporto. Antes de iniciar, verificamos se o aparelho estava funcionando corretamente ( Fig. 2 ) por meio de testes com objetos metálicos (cintos e celulares) e, sempre, os testes foram realizados sob a supervisão do técnico responsável para garantir o perfeito funcionamento.

**Fig. 1:** Detector de metais de passagem de aeroporto (CEIA SMD600 PLUS, modelo 2021).

**Fig. 2:** Teste de controle para certificar o funcionamento adequado do detector de arco usando celular no bolso.

Foram recrutados dois voluntários saudáveis, um homem (172 cm de altura) e uma mulher (156 cm de altura), que não possuíam nenhum dispositivo de metal em seu corpo. Os voluntários caminharam em duas velocidades diferentes cada um, um teste a 2 km/h e outro a 6 km/h. Inicialmente, como controle, os voluntários atravessaram o detector de metais sem implantes. Em seguida, fixamos os implantes ortopédicos aos voluntários ( Figs. 3 e 4 ), utilizando uma fita adesiva, respeitando ao máximo a verdadeira localização do implante no corpo. Todos os testes foram realizados duas vezes para ratificar os resultados da primeira passagem.

**Fig. 3:** Prótese total de joelho amarrada à superfície anterior do joelho direito antes do teste.

**Fig. 4:** Haste femoral retrógrada bilateral fixada na superfície lateral das coxas antes do teste.

Os implantes ( Tabela 1 ) utilizados para fixação de fraturas e para substituição articular foram avaliados em diferentes combinações (unilateral/bilateral, membro esquerdo/direito ou fraturas associadas) e sempre respeitando a localização anatômica (ombro, braço, cotovelo, antebraço, punho, quadril, coxa, joelho, perna e tornozelo). Os implantes para fixação interna foram fabricados pela Hexagon® (Itapira, SP, Brasil), Smith & Nephew (Memphis, TN, EUA) e Tóride (Mogi Mirim, SP, Brasil), o fixador externo pela Baumer (Mogi Mirim, SP, Brasil), as próteses de quadril da Baumer (Mogi Mirim, SP, Brasil) e Víncula (Rio Claro, SP, Brasil), e as próteses de joelho da Aesculap AG (Tuttlingen, Alemanha) e Baumer (Mogi Mirim, SP, Brasil).

**Tabela 1:** Conjuntos e combinações de implantes ortopédicos testados

Implante	Voluntário do sexo masculino				Voluntária do sexo feminino				Liga
	2 km/h		6 km/h		2 km/h		6 km/h		
	Teste 1	Teste 2	Teste 1	Teste 2	Teste 1	Teste 2	Teste 1	Teste 2	
Celular (controle)	P	P	P	P	P	P	P	P	−
Nenhum (controle)	N	N	N	N	N	N	N	N	-
Placa terço de tubo de pequenos fragmentos + 6 parafusos (unilateral)	N	N	N	N	N	N	N	N	Aço inoxidável NBR ISO 5832-1
Placa bloqueada de tíbia distal medial + 6 parafusos (unilateral)	N	N	N	N	N	N	N	N	Aço inoxidável NBR ISO 5832-1
Placa bloqueada de tíbia distal medial e anterolateral + 12 parafusos (unilateral)	N	N	N	N	N	N	N	N	Aço inoxidável NBR ISO 5832-1
Placa de calcâneo de pequenos fragmentos + 6 parafusos (unilateral)	N	N	N	N	N	N	N	N	Aço inoxidável NBR ISO 5832-1
Haste intramedular de tíbia + 4 parafusos de bloqueio (unilateral)	N	N	N	N	N	N	N	N	Aço inoxidável NBR ISO 5832-1
Haste intramedular retrógrada de fêmur + 3 parafusos de bloqueio (unilateral)	N	N	N	N	N	N	N	N	Aço inoxidável NBR ISO 5832-1
Haste intramedular retrógrada de fêmur + 3 parafusos de bloqueio (unilateral)	N	N	N	N	N	N	N	N	Aço inoxidável NBR ISO 5832-1
Haste intramedular retrógrada de fêmur bilateral + 6 parafusos de bloqueio + Haste intramedular de tíbia + 3 parafusos	N	N	N	N	N	N	N	N	Aço inoxidável NBR ISO 5832-1
Haste cefalomedular de fêmur + 1 parafuso deslizante de quadril + 2 parafusos de bloqueio (unilateral)	N	N	N	N	N	N	N	N	Titânio Ti-6Al-4V (ASTM F1472)
Haste cefalomedular de fêmur + 1 parafuso deslizante de quadril + 2 parafusos de bloqueio (bilateral)	N	N	N	N	N	N	N	N	Titânio Ti-6Al-4V (ASTM F1472)
DCS com parafusos (unilateral)	N	N	N	N	N	N	N	N	Aço inoxidável NBR ISO 5832-1
DHS com parafusos (unilateral)	N	N	N	N	N	N	N	N	Aço inoxidável NBR ISO 5832-1
Placa bloqueada de úmero proximal + 8 parafusos (unilateral)	N	N	N	N	N	N	N	N	Aço inoxidável NBR ISO 5832-1
Duas âncoras de 3,5 mm no ombro (unilateral)	N	N	N	N	N	N	N	N	Aço inoxidável NBR ISO 5832-1
Haste umeral intramedular + 3 parafusos de bloqueio (unilateral)	N	N	N	N	N	N	N	N	Aço inoxidável NBR ISO 5832-1
Placa bloqueada de clavícula + 6 parafusos (unilateral)	N	N	N	N	N	N	N	N	Aço inoxidável NBR ISO 5832-1
Placa bloqueada de clavícula + 6 parafusos (bilateral)	N	N	N	N	N	N	N	N	Aço inoxidável NBR ISO 5832-1
DCP 12 furos + 4 parafusos (úmero unilateral)	N	N	N	N	N	N	N	N	Aço inoxidável NBR ISO 5832-1
Dois parafusos canulados de 3,5 mm + fio de cerclagem (1,0 mm) (joelho unilateral)	N	N	N	N	N	N	N	N	Aço inoxidável NBR ISO 5832-1
Dois parafusos canulados de 3,5 mm + fio de cerclagem (1,0 mm) (joelho bilateral)	N	N	N	N	N	N	N	N	Aço inoxidável NBR ISO 5832-1
Dois parafusos canulados de 7,0 mm + 1 fio de cerclagem (1,0 mm) (joelho unilateral)	N	N	N	N	N	N	N	N	Aço inoxidável NBR ISO 5832-1
Dois parafusos canulados de 7,0 mm + 1 fio de cerclagem (1,0 mm) (joelho bilateral)	N	N	N	N	N	N	N	N	Aço inoxidável NBR ISO 5832-1
Placa bloqueada de rádio distal + 11 parafusos (unilateral)	N	N	N	N	N	N	N	N	Aço inoxidável NBR ISO 5832-1
Placa bloqueada de rádio distal + 11 parafusos (bilateral)	N	N	N	N	N	N	N	N	Aço inoxidável NBR ISO 5832-1
Placa bloqueada de rádio distal + 11 parafusos (unilateral)	N	N	N	N	N	N	N	N	Titânio (ASTM F-67)
Placa bloqueada de rádio distal + 11 parafusos (bilateral)	N	N	N	N	N	N	N	N	Titânio (ASTM F-67)
Prótese total primária cimentada de joelho (unilateral)	P	P	P	P	P	P	P	P	Cobalto-cromo-molibdênio (ISO 5832-4)
Prótese total primária cimentada de joelho (unilateral)	N	N	N	N	N	N	N	N	Cobalto-cromo-molibdênio (ISO 5832-4)
Prótese de quadril Thompson (unilateral)	P	P	P	P	P	P	P	P	Aço inoxidável NBR ISO 5832-1
Acetábulo não cimentado + haste femoral não cimentada (unilateral)	P	P	P	P	P	P	P	P	Titânio (ASTM F-67) (acetábulo) + Titânio Ti-6A-4V (ASTM F-136) e revestimento poroso de titânio (ASTM F-1580) (componente femoral)
Acetábulo não cimentado + haste femoral primária cimentada (unilateral)	P	P	P	P	P	P	P	P	Titânio (ASTM F-67) (acetábulo) + Aço inoxidável (NBR ISO 5832-9/ASTM F-1586)
Fixador externo (4 parafusos Schanz + 2 barras + 8 conectores barra-pino)	P	P	P	P	P	P	P	P	Aço inoxidável (ISO 5832-1, ASTM F-138) e liga de alumínio

Abreviações: ASTM, Sociedade Americana de Testes e Materiais; N, negativo; NBR ISO, Norma Brasileira Reguladora - International Organization for Standardization; P, positivo.

## Resultados

Os resultados são apresentados na Tabela 1 . De todos os materiais de osteossíntese testados, o fixador externo foi o único detectado e sua diferença em relação aos demais estava em sua composição – era de liga de alumínio. Todos os implantes de artroplastia do quadril testados foram detectados. Foram testados dois implantes de artroplastia do joelho, ambos feitos de cobalto-cromo-molibdênio, mas com especificações diferentes ( Tabela 1 ) e apenas um deles foi detectado.

## Discussão

Os pacientes frequentemente perguntam sobre aspectos práticos da vida diária e como certos tipos de cirurgia afetarão sua rotina e por quanto tempo. Muitas dessas questões ainda não possuem uma resposta definitiva baseada em estudos científicos. Uma pergunta extremamente comum é se os implantes ortopédicos retidos acionarão detectores de metais em aeroportos. Este tem sido um tema em alta desde 11 de setembro de 2001, quando o mundo ficou chocado com ataques terroristas em aviões, o que resultou no aumento de segurança em aeroportos para evitar tais atos. As principais preocupações geralmente são o inconveniente de ser revistado depois de acionar o detector de metais, a ansiedade de ser detido no aeroporto e a possibilidade de atrasar uma viagem ou perder um voo. Passados 21 anos dos ataques terroristas, desconhecemos a existência de um documento oficial e universalmente aceito que o paciente possa portar para provar a existência de um implante metálico ortopédico. Portanto, o procedimento padrão é uma triagem adicional se um indivíduo acionar o detector de metais de passagem, ainda que tenha consigo um laudo médico emitido pelo cirurgião ortopedista. 10 No Brasil, este é o primeiro estudo a investigar a detecção de implantes ortopédicos por detector de metais em aeroporto. Adicionalmente, todas as pesquisas publicadas existentes foram realizadas em detectores de arco com mais de 5 anos de fabricação, sendo que neste estudo foi utilizado um dispositivo de 2021, adotado universalmente em aeroportos internacionais. Vale ressaltar que este detector de metais atende integralmente o atual nível de segurança do Aeroporto Internacional Tancredo Neves (Confins), sob regulamentação da ANAC (Agência Nacional de Aviação Civil) e consequentemente da Organização Internacional de Aviação Civil.

Nossos achados foram completamente inesperados, pois nenhum dos conjuntos de osteossíntese foi detectado, exceto o fixador externo. Não foram observadas diferenças entre as duas velocidades distintas de trânsito avaliadas. Todos os testes foram realizados com implantes nos lados direito e esquerdo, para minimizar o potencial viés da distância do metal ao detector, e não foram observadas diferenças. Todos os conjuntos de osteossíntese testados foram confeccionados em Aço Inoxidável NBR ISO 5832-1, titânio F-67 (placa para rádio distal) e titânio Ti-6Al-4V (haste cefalomedular femoral), exceto o fixador externo que possuía liga de alumínio em sua composição. O único implante de aço inoxidável NBR ISO 5832-1 detectado foi a prótese de quadril Thompson, o que sugere que a concentração da massa do implante pode aumentar a detecção. Todos os outros tipos de prótese de quadril testados também dispararam o alarme. Um achado interessante foi a diferença na detecção entre os dois tipos de próteses totais de joelho testadas. Embora ambas as próteses de joelho fossem feitas de cobalto-cromo-molibdênio, a detectada foi fabricada no Brasil, enquanto a outra foi fabricada na Alemanha. Essa diferença entre as próteses de joelho testadas levanta uma suspeita sobre a composição da liga, que possivelmente poderia ter interferido no acionamento do detector de arco.

Considerando todas as combinações de implantes testadas e apresentadas na Tabela 1 , nosso estudo corrobora com os achados de Chan et al., 11 em que todos os pacientes utilizando somente implantes de pé e tornozelo passaram despercebidos. Este estudo está de acordo com vários outros da literatura, que afirmam que próteses totais articulares serão frequentemente detectadas, como as próteses de quadril em nosso estudo. 2 3 12 Kimura et al. 5 constataram que a taxa de detecção de implantes foi maior durante voos internacionais, o que pode explicar porque todas as próteses de quadril foram detectadas em nosso estudo, uma vez que o dispositivo utilizado foi definido para padrões internacionais, provavelmente uma configuração mais sensível.

Pesquisas anteriores sugeriram alguns fatores que podem influenciar na probabilidade de detecção, como massa do implante, combinações, composição, localização no corpo, lateralidade, velocidade de trânsito, modelo do detector, configurações de sensibilidade de acordo com o nível de segurança do aeroporto e mascaramento de tecido. 4 8 11 Em nosso estudo, apenas pudemos observar a provável interferência da massa, densidade e material do implante.

Uma informação interessante sobre os detectores de metais em aeroportos é o fato de que eles registram cada indivíduo que passa pelo arco, mesmo que não haja nenhum dispositivo de metal in situ. Esses dados são usados em um software interno de randomização, que aciona um alarme sonoro para selecionar um passageiro aleatório para ser revistado minuciosamente pela segurança do aeroporto. Este alarme sonoro é diferente do som padrão acionado quando um dispositivo metálico é identificado. Esse mecanismo de seleção aleatória de passageiros a serem revistados pode ser a razão pela qual alguns pacientes com implantes ortopédicos relatam ter sido revistados, o que é um possível viés de pesquisas anteriores usando informações retrospectivas. 8

As limitações do nosso estudo incluem o fato de que nenhum dos implantes ortopédicos estava dentro dos voluntários testados e ainda não sabemos se uma interação osso-implante ou tecido mole-implante afetaria a detecção. Pesquisas anteriores tentaram avaliar como o envelope de tecido mole afeta a detecção de metais, porém os resultados foram conflitantes. 4 11 Outra limitação é que não conseguimos testar todos os implantes ortopédicos disponíveis. No entanto, julgamos que foram avaliadas as situações mais frequentes. Nosso estudo também não avaliou a detecção por detector de metais portátil, que supostamente é mais sensível. Nossas conclusões foram baseadas no atual nível de segurança do Aeroporto Internacional de Confins. Se, por algum motivo, o nível de segurança do aeroporto aumentar, as configurações do dispositivo também serão alteradas e a sensibilidade de detecção será aprimorada. Embora os testes tenham sido realizados em ambiente real de um aeroporto internacional, seguindo todos os requisitos de segurança e sob as normas da ANAC e ICAO, não podemos afirmar que nossos achados serão fielmente reprodutíveis se realizados em outros aeroportos, utilizando um dispositivo detector diferente ou um nível de segurança diferente.

No entanto, alguns pontos relevantes merecem ser destacados. Este é o primeiro estudo realizado no Brasil com o objetivo de esclarecer informações sobre a detecção de implantes ortopédicos em um aeroporto. Fornecemos informações úteis aos pacientes, pois os implantes para fixação interna provavelmente não serão detectados em condições normais. Por outro lado, provavelmente serão detectados fixadores externos e próteses. Também tentamos minimizar possíveis vieses observados em estudos semelhantes. Testamos diferentes composições metálicas em dois voluntários saudáveis, sob duas velocidades diferentes para passagem pelo arco do detector, com implantes fixados em ambos os lados (direito e esquerdo) do corpo, para minimizar o viés da distância do detector ao implante.

## Conclusões

Neste estudo com implantes ortopédicos ex-vivo, verificamos que os implantes de osteossíntese para fixação interna, compostos por Aço Inox ISO 5832-1 e titânio, não foram detectados pelo detector de metais do aeroporto e não devem causar transtornos aos pacientes durante a viagem. Entretanto, fixadores externos e próteses articulares totais serão detectados com mais frequência.

É importante destacar as limitações do nosso estudo e a necessidade de aprofundar esta questão incluindo implantes ortopédicos in vivo e testando próteses com diferentes tamanhos, pesos e materiais.
